# Supplementary material for: Association of Obesity and Malnutrition with In-Hospital Mortality and Clinical Outcomes in Patients Receiving Maintenance Dialysis: A National Database Study
Source: Nutrients. 2026 Jan 2;18(1):157. doi: 10.3390/nu18010157 (PMC12787828; doi:10.3390/nu18010157)
Supplement: Supplementary file 1 [file nutrients-18-00157-s001.zip › nutrients-4011449-supplementary.pdf]

Table S1. Corresponding ICD-10 codes for diagnoses and procedures

Diagnosis	ICD 10 codes
Malnutrition	Mild to moderate; E44.0, E44.1, E46<br>Severe; E40, E41, E42, E43, R64
Obesity	E66.0—Obesity due to excess calories, E66.09—Other obesity due to excess calories, E66.1—Drug-induced obesity, E66.2—Morbid obesity with alveolar hypoventilation, E66.8—Other obesity, E66.9—Obesity, unspecified
ESRD	N185, N186
Acute kidney injury	N170, N171, N172, N178, N179, N19, N990, O904
Peritoneal dialysis	3E1M39Z
Hemodialysis	5A1D70Z, 5A1D80Z, 5A1D90Z
Kidney transplantation	Z94.0
Diabetes mellitus	E10.0, E10.1, E10.6, E10.8, E10.9,<br>E11.0, E11.1, E11.6, E11.8, E11.9,<br>E12.0, E12.1, E12.6, E12.8, E12.9,<br>E13.0, E13.1, E13.6, E13.8, E13.9,<br>E14.0, E14.1, E14.6, E14.8, E14.9<br>E10.2–E10.5, E10.7, E11.2–E11.5, E11.7, E12.2–E12.5, E12.7, E13.2–E13.5, E13.7, E14.2–E14.5, E14.7
Hypertension	I10.X
Coronary artery disease	I2510, I7090, I1779, I519, I259, I700, I672, I652, I25798, I25791, I25709, I25708, I25700, I25701, I2584, I2583, I25119, I25118, I25110, I25111, I25119, I25810, I7209, I25811, I25759, I25750, I25751, I25759, I70209, K511, I78, I7090, I672, G9519, I270
Smoking	Z87891, Z720, Z5301, Z716
Alcohol use	F1012, F1020, F1090, F1022, F1092
Volume overload	E8770, E8771, E8779
Sepsis	A409, A412, A4101, A411, A403, A414, A4151, A4152, A4153, A4159, T80219A, T80211A, T80212A, T8022XA, T8029XA, K6811, N390

Catheter-related blood stream infection	T80.211A
Congestive heart failure	I09.9, I11.0, I13.0, I13.2, I25.5, I42.0, I42.5–I42.9, I43.x, I50.x, P29.0
Peripheral vascular disease	I70.x, I71.x, I73.1, I73.8, I73.9, I77.1, I79.0, I79.2, K55.1, K55.8, K55.9, Z95.8, Z95.9
Cerebrovascular disease	G45.x, G46.x, H34.0, I60.x–I69.x
Dementia/cognitive impairment	F00.x–F03.x, F05.1, G30.x, G31.1
Moderate or severe liver disease	I85.0, I85.9, I86.4, I98.2, K70.4, K71.1, K72.1, K72.9, K76.5, K76.6, K76.7
Diabetes without chronic complication	E10.0, E10.1, E10.6, E10.8, E10.9, E11.0, E11.1, E11.6, E11.8, E11.9, E12.0, E12.1, E12.6, E12.8, E12.9, E13.0, E13.1, E13.6, E13.8, E13.9, E14.0, E14.1, E14.6, E14.8, E14.9
Diabetes with chronic complication	E10.2–E10.5, E10.7, E11.2–E11.5, E11.7, E12.2–E12.5, E12.7, E13.2–E13.5, E13.7, E14.2–E14.5, E14.7
Any malignancy, including lymphoma and leukemia, except malignant neoplasm of skin	C00.x–C26.x, C30.x–C34.x, C37.x–C41.x, C43.x, C45.x–C58.x, C60.x–C76.x, C81.x–C85.x, C88.x, C90.x–C97.x
Metastatic solid tumor	C77.x–C80.x
**Procedure**	**ICD-10 codes**
Blood transfusion	30233N1, 30243N1
Need for vasopressors	3E033XZ
TPN use	3E0336Z, 3E0436Z
Mechanical ventilation	5A0935Z, 5A0955Z, 5A09357, 5A09358, 5A09359, 5A0935A, 5A0935B, 5A09457, 5A09458, 5A09459, 5A0945A, 5A0945B, 5A09557, 5A09558, 5A09559, 5A0955A, 5A0955B, Z991, Z9910, Z9911, Z9912

Table S2. The association between obesity with/without malnutrition (vs non-obese) and in-hospital mortality, adverse clinical outcomes, inpatient treatments and resource utilization in ESKD patients stratified by dialysis modality (HD vs PD).

In-hospital outcomes	Non-obese	PD Adjusted OR† (95% CI) Obesity w/o malnutrition	P-value	PD Adjusted OR† (95% CI) Obesity + malnutrition	P-value	HD Adjusted OR† (95% CI) Obesity w/o malnutrition	P-value	HD Adjusted OR† (95% CI) Obesity + malnutrition	P-value	P for interaction†
In-hospital mortality	Ref.	0.72 (0.61–0.86)	<0.001	2.05 (1.37–3.09)	0.001	0.88 (0.69–1.13)	0.323	2.10 (1.17–3.77)	0.013	0.083
Sepsis	Ref.	1.09 (1.02–1.17)	0.008	2.28 (1.87–2.78)	<0.001	1.07 (1.05–1.09)	<0.001	2.68 (2.53–2.82)	<0.001	0.220
CRBSI	Ref.	1.40 (0.89–2.19)	0.147	2.88 (0.91–9.18)	0.073	1.09 (1.03–1.15)	0.005	1.70 (1.44–2.00)	<0.001	0.400
Volume overload	Ref.	1.29 (1.17–1.42)	<0.001	1.37 (0.97–1.93)	0.071	1.07 (1.05–1.10)	<0.001	0.89 (0.81–0.97)	0.009	<0.001
Need for vasopressors	Ref.	0.99 (0.79–1.24)	0.936	1.90 (1.07–3.38)	0.028	1.07 (1.02–1.13)	0.010	2.67 (2.37–3.02)	<0.001	0.439
TPN use	Ref.	0.50 (0.27–0.93)	0.028	1.97 (0.63–6.19)	0.246	0.58 (0.50–0.67)	<0.001	4.70 (3.89–5.68)	<0.001	0.314
Mechanical ventilation	Ref.	2.21 (1.95–2.51)	<0.001	1.53 (0.94–2.48)	0.088	1.83 (1.78–1.89)	<0.001	1.98 (1.81–2.17)	<0.001	0.007
Blood transfusion	Ref.	0.91 (0.83–0.99)	0.030	1.49 (1.14–1.95)	0.003	0.89 (0.86–0.91)	<0.001	1.62 (1.51–1.74)	<0.001	0.719
	**Non-obese**	**PD Adjusted coefficient (Δ, 95% CI) Obesity w/o malnutrition**	**P-value**	**PD Adjusted coefficient (Δ, 95% CI) Obesity + malnutrition**	**P-value**	**HD Adjusted coefficient (Δ, 95% CI) Obesity w/o malnutrition**	**P-value**	**HD Adjusted coefficient (Δ, 95% CI) Obesity + malnutrition**	**P-value**	**P for interaction†**
LOS (days)	Ref.	−0.48 (−0.66 to −0.29)	<0.001	+3.70 (+2.72 to +4.68)	<0.001	+0.29 (+0.23 to +0.36)	<0.001	+7.16 (+6.72 to +7.61)	<0.001	<0.001
Hospitalization cost ($)	Ref.	−5,270.73 (−8,165.68 to −2,375.78)	<0.001	+48,139.76 (+32,881.62 to +63,397.91)	<0.001	+5,615.18 (+4,399.77 to +6,830.59)	<0.001	+101,968.50 (+93,591.12 to +110,345.90)	<0.001	<0.001

Abbreviation: CRBSI, catheter related bloodstream infection; HD; hemodialysis; LOS, length of stay; PD, peritoneal dialysis; TPN, total parenteral nutrition.

*Adjusted estimates from multivariable models controlling prespecified covariates (e.g., age, gender, race, hospitalization year, Charlson comorbidity score, diabetes mellitus, congestive heart failure, hypertension, coronary artery disease, cerebrovascular disease, peripheral vascular disease, cancer, cirrhosis dementia/cognitive impairment, alcohol drinking, smoking, mode of KRT and admission type, hospital location/ teaching status).

†P for interaction obtained from adding a product term between dialysis modality (HD vs PD) and obesity/malnutrition category (3-level exposure) in the fully adjusted model (overall interaction test, typically 2 df).
Ref. = Non-obese within each dialysis modality stratum.
